# Weakly supervised multiple-instance active learning for tooth-marked tongue recognition

**DOI:** 10.3389/fphys.2025.1598850

**Published:** 2025-06-11

**Authors:** Feilin Deng, Shangxuan Li, Zizhu Yang, Wu Zhou

**Affiliations:** School of Medical Information Engineering, Guangzhou University of Chinese Medicine, Guangzhou, China

**Keywords:** tooth-marked tongue, weakly supervised learning, multiple instance learning, active learning, pseudo label

## Abstract

**Introduction:**

Recognizing a tooth-marked tongue has important clinical diagnostic value in traditional Chinese medicine. Current deep learning methods for tooth mark detection require extensive manual labeling and tongue segmentation, which is labor-intensive. Therefore, we propose a weakly supervised multipleinstance active learning model for tooth-marked tongue recognition, aiming to eliminate preprocessing segmentation and reduce the annotation workload while maintaining diagnostic accuracy.

**Method:**

We propose a one-stage method tongenerate tooth mark instances that eliminates the need for pre-segmentation of the tongue. To make full use of unlabeled data, we introduce a semisupervised learning paradigm to pseudo-label unlabeled tongue images with high model confidence in active learning and incorporate them into the training set to improve the training efficiency of the active learning model. In addition, we propose an instance-level hybrid query method considering the diversity of tooth marks.

**Result:**

Experimental results on clinical tongue images verify the effectiveness of the proposed method, which achieves an accuracy of 93.88% for tooth-marked tongue recognition, outperforming the recently introduced weakly supervised approaches.

**Conclusion:**

The proposed method is effective with only a small amount of image-level annotation, and its performance is comparable to that of image-level annotation, instance-level annotation and pixel-level annotation, which require a large number of tooth markers. Our method significantly reduces the annotation cost of the binary classification task of traditional Chinese medicine tooth mark recognition.

## 1 Introduction

The tongue is the main organ of human internal organs and can reflect disorder and even pathological changes in human internal organs. Tongue diagnosis is a non-invasive diagnostic method that provides important signs for early diagnosis of disease and human health. The tooth mark, one of the most important features of the tongue, is usually formed when the tongue body is squeezed by adjacent teeth. The clinical manifestations of patients with a tooth-marked tongue are anorexia, abdominal distention, gastric distention, and constipation (David Zhang and Zhang, 2017). Therefore, identifying tooth-marked tongue is of great value for clinical diagnosis. In the clinic, tooth-marked tongue recognition depends on doctors’ subjective observation of the morphological information of the tooth marks on the tongue. Researchers have attempted to use digital image processing and feature analysis for objective tooth mark recognition, especially based on tooth mark shape and color features (Hsu et al., 2011; Lo et al., 2012; Shao et al., 2014). However, due to the great differences in the shape and color of tooth marks, it is difficult to ensure the reliability and accuracy of tooth-marked tongue recognition according to the color or shape characteristics.

Deep neural networks (DNNs), with their significant feature representation advantages, have been applied to tooth-marked tongue recognition. There are two kinds of tooth-marked tongue recognition methods: one is tooth-marked tongue classification based on supervised image-level annotation, and the other is object detection based on tooth-marked areas. For the first category of tooth-marked tongue image classification, Sun et al. (2019) proposed to classify tooth-marked tongue images by deep convolution networks, and the concerned area of tooth marks is visualized by the Grad-CAM (Selvaraju et al., 2017) model. Wang et al. (2020) used a deeper convolutional neural network (CNN), Resnet34 (He et al., 2016), and demonstrated that their method achieved better efficiency and scene generalization ability. Lu et al. (2023) proposed a prior regularization tooth-marked tongue recognition method utilizing the prior knowledge of the location and width of tooth marks. Tan et al. (2023) used a non-subsampled wavelet transform for multi-scale decomposition and applied the autoregressive local linear model encoding algorithm to retain key texture information and remove redundant data in the decomposed sub-images. This enabled the model to more comprehensively extract the texture features of tooth-marked tongues, thereby improving the accuracy of identification.

However, tooth-marked tongue recognition is a fine-grained classification problem, which is not suitable for classification using the above image-level supervision information (Fu et al., 2017). For the other category of tooth-marked tongue recognition with respect to object detection, Weng et al. (2021) proposed that the tooth mark on the tongue be selected by frame, introduced the YOLOv3 (Redmon and Farhadi, 2018) model in object detection to detect the tooth mark, and obtained promising results. Li et al. (2018) considered tooth-marked tongue recognition as a multiple instance learning (MIL) framework and first used the prior knowledge that the tooth mark is concave–convex to generate candidate regions. They then trained an instance feature extractor followed by multi-instance classification Via support vector machine (MISVM) (Andrews et al., 2002) for classification to obtain good performance. The above methods based on object detection can yield promising performance, but they require a large number of tooth mark instances with strong annotation, which carries a huge labor cost. Recently, Zhou et al. (2022) proposed a weakly supervised target detection model (WSDDN) (Bilen and Vedaldi, 2016) for tooth-marked tongue recognition to avoid the labor cost of toothmark instances. However, this method requires tongue segmentation in advance, which has a very large clinical workload. More importantly, the above tooth-marked tongue recognition methods do not consider large numbers of unlabeled data. When clinical labeling is very cumbersome, this unlabeled tongue information should be included to improve the performance of tooth-marked tongue recognition.

Tooth-marked tongue detection with few image-level annotations is a very challenging problem. First, if the tongue body is not pre-segmented, the boundary information of the tongue cannot be obtained. Consequently, it becomes difficult to extract samples based on the prior knowledge that tooth marks are distributed on the edge of the tongue body. Then, the simple instance generation method (Van de Sande et al., 2011; Girshick, 2015; Zitnick and Dollár, 2014) in object detection often generates many instances from the background so that the image-level information learned by the model is seriously disturbed by background noise, resulting in inconsistent uncertainty between image-level and target instances. Subsequently, a large amount of unlabeled tongue image data with tooth mark information is not utilized by deep learning networks, and whether to use active learning or semi-supervised learning for fine-grained detection tasks without instance-level labeling is still an unexplored problem. Finally, due to the diversity of the instances of tooth marks produced by the tooth-marked tongue, it is difficult to accurately represent image-level information only by the uncertainty of the instance.

In this work, we propose a weakly supervised multiple-instance active learning (WSMIAL) model for tooth-marked tongue recognition that can significantly reduce the labeling cost from three aspects: instance-level tooth mark region selection, pixel-level tongue segmentation, and image-level category labeling. Specifically, we propose a one-stage method to generate toothmark instances without pre-tongue segmentation. Then, we introduce a semi-supervised learning paradigm to pseudo-label images with high model confidence and incorporate them into the training set to improve the training efficiency of the active learning model. In addition, we align the uncertainty consistency between the tooth mark instances and the tongue image and propose an instance-level hybrid query method considering the diversity of tooth marks. Through comparative experiments of clinical tongue images with related work, the proposed model has a competitive performance in tooth-marked tongue recognition, and its annotation cost is much lower than the existing tooth-marked tongue recognition models.

## 2 Materials and methods

### 2.1 Study population and tooth mark labels

**FIGURE 1:**
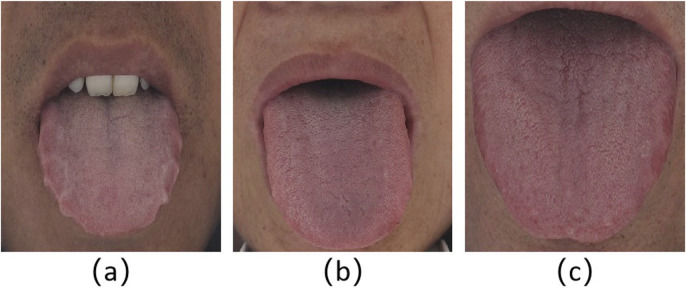
Representative tongue images. **(a)** Tooth-marked tongue. The outline of both sides of the tongue is obviously distorted, and the color of the tongue extrusion area changes; **(b)** tongue without tooth marks. The tongue is flat, with regular peripheral contour, without contour deformation and discoloration area; **(c)** suspicious tooth-marked tongue. Because the tongue body is flat and the surrounding contour is not distorted, its label is finally determined as a tooth-marked tongue by the color change of the extrusion area of the tongue edge.

The study was approved by the local ethics committee, and the patients signed the informed consent form. We used standard equipment designed by Daoshi Medical Technology Co., Ltd. (DS01-B) to obtain tongue images from patients in the local institute. We obtained 1,108 tongue images from the local medical research institution. Figure 1 shows the representative tongue images with or without tooth marks. The clinical criteria for the diagnosis of tooth-marked tongue in traditional Chinese medicine are as follows: first, observe whether there are teeth squeezing on both sides of the tongue body, resulting in tooth marks; Second, for parts of the tongue where the tooth mark is not obvious, observe the color depth of the suspicious area. The extruded tooth mark area usually has a darker color (Weng et al., 2021), as shown in Figures 1a,c. Four traditional Chinese medicine doctors with 2–5 years of clinical experience divided the tongue image into tooth-marked or non-tooth-marked areas and framed the mark area judged as a tooth-marked tongue to construct the tongue image data set. Although the proposed method in this work does not need to segment the tongue in advance and does not need instances of tooth marks to realize other relevant tooth-marked tongue recognition methods and compare their performance, we also arranged for doctors to segment the tongue and label the tooth marks.

**FIGURE 2:**
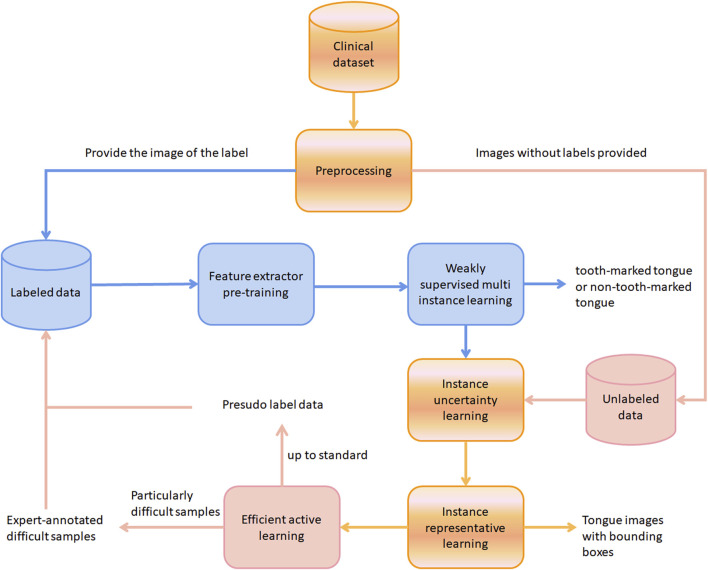
The workflow of this work. The blue, light pink, and black arrows represent the processes of labeled data, unlabeled data, and combined data processing, respectively.

The flowchart of the work in this article is presented in Figure 2. The blue arrows represent the workflow of labeled data, the light pink arrows represent the workflow of unlabeled data, and the gradient arrows represent the workflow of sharing labeled and unlabeled data. Specifically, during the pre-training of the feature extractor and the weakly supervised multi-instance learning stage, only the data with labels is used, and the output is a binary classification label of whether an image represents a toothed tongue or not. In the instance uncertainty learning stage, unlabeled data are added to the training, and then they enter the instance representation learning stage together. The output is a tongue image with a bounding box of the predicted tooth mark area. Finally, the unlabeled data are subjected to efficient active learning. The pseudo-labeled standard samples are finally placed in the label data pool to start a new round of training. Only samples that are particularly difficult to identify will be given to the experts for annotation.

### 2.2 Proposed method

In order to reduce the interference of the background on tooth mark recognition, previous studies (Li et al., 2018; Sun et al., 2019; Wang et al., 2020; Weng et al., 2021; Zhou et al., 2022) generally needed to preliminarily segment the tongue, which has high labor costs. By comparison, we use a one-stage detection method to directly extract the candidate tooth mark area to avoid the tedious work of tongue pre-segmentation. Then, considering that tooth-marked tongue recognition is a fine-grained classification problem, we adopt the multi-instance learning paradigm (Li et al., 2018). In addition, obtaining a large number of labeled tooth-marked tongues requires high labor costs, while unlabeled data are relatively easy to obtain and have not been used in tooth-marked tongue recognition. Typically, there are two ways to use unlabeled data: active learning and semi-supervised learning. The former requires expert participation, and the latter does not. Inspired by the works by Wang et al. (2017) and Zhang et al. (2022), we use the combination of the two to obtain better performance. The main difference is that we acquire image information based on instance information and build a multi-instance efficient active learning framework.

Previous studies have shown that weakly supervised target detection can assist in locating the location of tooth marks, and its advantage is that it does not require instance-level annotation (Zhou et al., 2022). If the active learning model is introduced based on the weakly supervised network, a significant reduction of instance-level and image-level annotations can be achieved. However, there are two problems in simply combining weakly supervised target detection and active learning. The first is weak supervision. There is no instance box labeling, so it is difficult to filter instances. If the image information is simply obtained by averaging the instance information with high uncertainty, it will lead to the problem of background instance interference. Second, due to the diversity of tooth marks, the selected examples cannot fully represent the image. In order to solve the above problems, we start with the uncertainty consistency between instances and images and the representative learning of instances and get the best image-level information through multi-instance learning.

**FIGURE 3:**
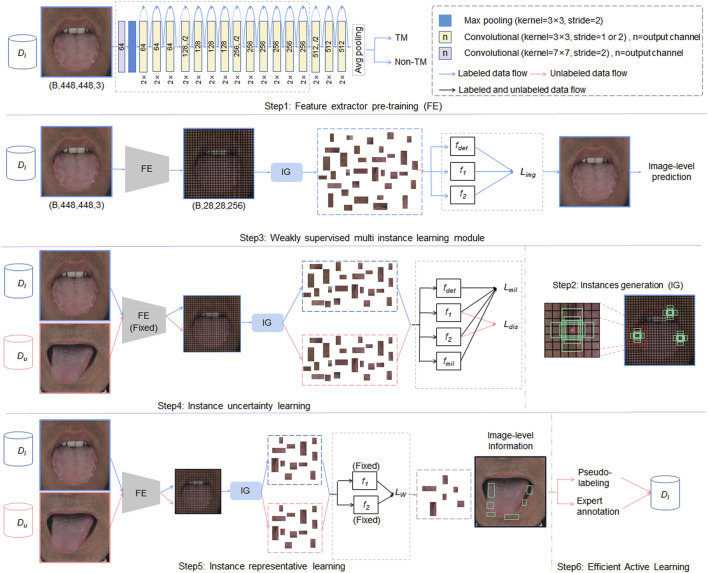
The proposed weakly supervised multiple-instance active learning (WSMIAL) network. FE means feature extractor, $D_{u}$ means unlabeled data pool, $D_{l}$ means labeled data pool, and $D_{pl}$ means pseudo-label data pool (best viewed in color).

Figure 3 shows the overall framework of the proposed WSMIAL model. The framework is composed of an instance generation module, a weakly supervised learning module, and a multi-instance active detection module.

As shown in Figure 3, the blue, red, and black arrows represent the processes of labeled data, unlabeled data, and combined data processing, respectively. Initially, in step 1, the feature extractor is pre-trained using labeled data. Subsequently, step 2 involves generating instance proposals, which are then employed in step 3 for image-level predictions aimed at identifying tooth-marked tongues. Step 4 performs instance uncertainty learning, fixes the feature extractor’s parameters, and fine-tunes the instance classifiers ( $f_{\det}$ , $f_{1}$ , $f_{2}$ , $f_{\mathrm{mil}}$ ) to maximize prediction discrepancies on unlabeled data, thereby highlighting high-uncertainty instances. Following this, step 5 shifts focus to instance-representative learning. The classifiers’ parameters are fixed, and instead, the feature extractor is fine-tuned to enhance its ability to select the most representative instances, ultimately outputting the object detection results. Finally, step 6 leverages active learning to select images that meet the pseudo-labeling criteria and incorporates them into the training dataset. Refer to Algorithm 1 for the specific process. Each module is detailed in the following sections.

Algorithm 1 Process of WSMIAL.

Input: Test set data pool $D_{t}$ , unlabeled data pool $D_{u}$ , labeled data pool $D_{l}$ , pseudo-labeled data pool $D_{pl}$ , number of selected samples K , number of selected instances $R_{n}$ , pseudo-labeling threshold δ , threshold decay rate dr , maximum number of active learning iteration A , and times of fine-tuned epoch .
1: a=0 , pre-train FE $\leftarrow \;D_{l}$

2: while a<A **do**
3: Fine-tuned model, $L_{\mathrm{img}}\;\leftarrow \;y_{cr}\;\leftarrow \;y^{f_{1}}$ , $y^{f_{2}}$ , $y^{f_{\det}}\;\leftarrow \;D_{l}$ , $D_{pl}$ , epoch

4: Remove pseudo-annotation, $D_{u}\;\leftarrow \;D_{pl}$

5: Use instance information to select K image samples:
6:  Fixed FE
7: $R_{n}*2$ instances $\leftarrow \;L_{\mathrm{mil}}$ , $L_{\mathrm{dis}}\;\leftarrow \;y^{f_{1}}$ , $y^{f_{2}}$ , $y^{f_{\mathrm{mil}}}$ , $y^{f_{\det}},D_{u}$ , $D_{l}$ , $R_{n}$ , epoch/2

8:  Unfixed FE, fixed $f_{1}$ , $f_{2}$

9: $R_{n}$ instances ← , $L_{w}\;\leftarrow \;y^{f_{1}}$ , $y^{f_{2}}$ , $D_{u}$ , $D_{l}$ , $R_{n}$ , epoch/2

10:  Top K← Image information $\leftarrow \;R_{n}$ instances
11:  Unfixed $f_{1}$ , $f_{2}$

12: Expert annotation, $D_{l}\;\leftarrow \;D_{u}$ , K

13: Pseudo-annotation, $D_{l}\;\leftarrow \;D_{pl}\;\leftarrow \;D_{u}$ , δ

14: Threshold decay, δ←a , dr

15: a=a+1

16: end while
17: Test model $\leftarrow \;D_{t}$

Output: Image-Level Prediction

### 2.3 Instances generation module

Similar to Weng et al. (2021), we use a one-stage method to generate instances of tooth marks. The preset boundary boxes are the length and width obtained by K-means clustering (Redmon and Farhadi, 2018), while the backbone of feature extraction is replaced by Resnet34, which has been demonstrated to be the best tooth mark feature extraction model (Wang et al., 2020). For the predefined bounding boxes, we randomly select nine clusters and three scales, evenly distributing the clusters across the scales. The nine clusters are (37, 19), (20, 41), (25, 36), (37, 35), (27, 52), (21, 69), (34, 47), (37, 64), and (32, 90). We divide images into S×S grid cells. If the center of a target falls into a grid, the grid is responsible for detecting the object. Each grid cell predicts the bounding boxes and the confidence score of the boxes. The size of S depends on the size of the input image size and the size of the downsampling. S is calculated by dividing the input image size by the downsampling factor of the network.

### 2.4 Weakly supervised learning module

In the instance generation module, each lattice is responsible for predicting the bounding box and the confidence of the bounding box, but it may not completely contain objects. The generated bounding box may not be able to frame the target well, and there is no real bounding box, so the confidence cannot be calculated according to the definition (Redmon et al., 2016): $confidence=pred\left(object\right)*IOU_{\mathrm{pred}}^{\mathrm{truth}}$ . Therefore, we follow the method of Zhou et al. (2022), using the score of the detection branch to describe the confidence of the bounding box, and use the detection score and classification score to calculate the image-level label and weakly monitor the accuracy of frame selection through the image-level loss (Zhou, 2017).

Specifically, the detection branch is defined as Equation 1:

$$y_{cr}^{\det}=\frac{e^{x_{cr}^{d}et}}{\sum_{r=1}^{R_{n}}e^{x_{cr}^{d}et}}$$ (1)

where $y_{cr}^{\det}\in R^{C\times R_{n}}$ is the prediction score of all regions in a category. c represents the category, and r represents the region.

The classification branch is defined as Equations 2, 3:

$$y_{\mathrm{crt}}^{\mathrm{cls}}=\frac{\sum_{t=1}^{T}x_{\mathrm{crt}}^{\mathrm{cls}}}{T}$$ (2)

$$y_{cr}^{\mathrm{cls}}=\frac{e^{y_{\mathrm{crt}}^{\mathrm{cls}}}}{\sum_{c=1}^{C}e^{y_{\mathrm{crt}}^{\mathrm{cls}}}}$$ (3)

where $y_{\mathrm{crt}}^{\mathrm{cls}}\in R^{C\times R_{n}}$ is the predicted score of all categories in a candidate area, and t represents the number of branches.

In the two branches of our model, the final score of each region is obtained by taking the Hadamard product of two module scores:

$$y_{cr}=\overset{R_{n}}{\underset{r=1}{\sum }}y_{cr}^{\mathrm{cls}}▪y_{cr}^{\det}$$ (4)

Finally, we calculate the image classification loss function as Equation 5:

$$L_{\mathrm{img}}\left(x\right)=\overset{C}{\underset{c=1}{\sum }}y^{GT}▪log\left(y_{cr}\right)$$ (5)

### 2.5 Multi-instance active learning

The key to active learning lies in sample selection. If we simply use the image score in Equation 4 as the information degree of the image to select the sample, we will inevitably introduce many background noise instances. Because we do not use the pre-segmentation method to generate candidate instances, the image score will be calculated using many background noises. In addition, there is diversity in tooth mark instances, and such an image score cannot represent the information degree of the image. Therefore, we build the instance uncertainty learning module and the instance representative learning module.

#### 2.5.1 Instance uncertainty learning

To learn the uncertainty at the instance level, we use two antagonistic instance classifiers in the detection network to obtain the prediction of the instance (Lakshminarayanan et al., 2017). The prediction difference between these two classifiers is maximized to predict the uncertainty of the instance. The relevant equations are shown in Equations 6, 7:

$$L=\underset{x\in D_{l}}{\sum }L_{\mathrm{img}}\left(x\right)-\underset{x\in D_{u}}{\sum }\beta ▪L_{\mathrm{dis}}\left(x\right)$$ (6)

$$L_{\mathrm{dis}}\left(x\right)=\overset{Rn}{\underset{r=1}{\sum }}|y_{cls1\text{\_}cr}-y_{cls2\text{\_}cr}|$$ (7)

where $y_{cls1\text{\_}cr},y_{cls2\text{\_}cr}\in R^{Rn\times C}$ are the instance classification predictions of the $r^{th}$ instance in image x by two classifiers.

In addition, we add multiple instance learning classifiers $f^{\mathrm{mil}}$ in parallel next to the instance classifiers to reweight the uncertainty of the instances, and the calculation process of the classification score in Equation 3 of multiple instances in the same image is updated as

$$y_{cr}^{\mathrm{mil}}=\frac{e^{x_{cr}^{\mathrm{mil}}}}{\sum_{c=1}^{C}e^{x_{cr}^{\mathrm{mil}}}}▪\frac{e^{y_{\mathrm{crt}}^{\mathrm{cls}}}}{\sum_{c=1}^{C}e^{y_{\mathrm{crt}}^{\mathrm{cls}}}}$$ (8)

The classification score Equation 4 at the image level is updated to Equation 9

$$y_{cr}^{*}=\overset{R_{n}}{\underset{r=1}{\sum }}y_{cr}^{\mathrm{mil}}▪y_{cr}^{\det}$$ (9)

We reweight the uncertainty score of instances by minimizing the loss of image classification to reweight the uncertainty of instances while filtering noise instances. This actually defines an expectation maximization procedure (Andrews et al., 2002; Bilen and Vedaldi, 2016). Considering that the MIL score of instances from backgrounds is very small, this can be achieved by optimizing the following loss function:

$$L_{\mathrm{mil}}\left(x\right)=\overset{C}{\underset{c=1}{\sum }}y^{GT}▪log\left(y_{cr}^{*}\right)$$ (10)

This makes instances with larger classification scores but smaller MIL scores be suppressed as the background. First, the initial detector is obtained by applying MIL loss in the labeled pool training process, and then the instance uncertainty in the unlabeled set is reweighted. We combine the image classification scores of all categories into a score vector, and then reweight the example uncertainty, as represented in Equation 11:

$$L_{\mathrm{dis}}\left(x\right)=\overset{R_{n}}{\underset{r=1}{\sum }}|w_{r}|▪\left(y_{cls1\text{\_}cr}-y_{cls2\text{\_}cr}\right)$$ (11)

Finally, we update the optimization loss in Equation 6 to Equation 12:

$$L=\underset{x\in D_{l}}{\sum }\left(L_{\mathrm{img}}\left(x\right)+L_{\mathrm{mil}}\left(x\right)\right)-\underset{x\in D_{u}}{\sum }\beta ▪L_{\mathrm{dis}}\left(x\right)$$ (12)

According to Equation 8 and Equation 10, MIL loss ensures that the highlighted instance uncertainty can represent the image uncertainty, that is, minimizing the classification loss of the image and minimizing the gap between the instance uncertainty and the image uncertainty. Through the iterative optimization of Equation 12, the gap between instance-level observation and image-level evaluation can be narrowed statistically, and this is helpful in suppressing instances with high noise.

#### 2.5.2 Instance representative learning

A well-known problem of uncertainty-based sampling in active learning is the so-called sampling bias (Li et al., 2021), indicating that the current instance cannot represent the image’s potential distribution (Yuan et al., 2021). In particular, during the generation of tooth marks, there are many instances with similar characteristics to tooth marks that are not distributed on the tongue. As shown in Figure 3, teeth, lips, cracks, and ecchymosis are distributed on the tongue, which makes the instances based on uncertainty alone unable to fully represent the information of the image. Therefore, we introduce Wasserstein distance to select the most representative instances.

The advantage of Wasserstein distance (Villani, 2009) is that even if the two distributions do not overlap, Wasserstein distance can still reflect their distance. This has been applied to the field of image generation and has been demonstrated to be a good measure of diversity (Gulrajani et al., 2017; Zhang et al., 2020). The Wasserstein distance is defined as

$$W\left(P_{L},P_{U}\right)=\underset{\gamma ∼II\left(P_{L},P_{U}\right)}{inf}E_{\left(m,n\right)∼\gamma }\left[\left\|m-n\right\|\right]$$ (13)

where $\left(P_{L},P_{U}\right)$ is the set of all possible joint distributions of $P_{L}$ and $P_{U}$ . Conversely, the marginal distributions of each distribution are $P_{L}$ and $P_{U}$ . Each possible joint distribution can be sampled from (m,n)∼γ to get two instance samples m , n and calculate the distance of the pair of samples, so the expected value of the distance of the samples under the joint distribution can be calculated as $E_{\left(m,n\right)∼\gamma }\left[\left\|m-n\right\|\right]$ . The lower bound that can be obtained for this expected value in all possible joint distributions is defined as the Wasserstein distance.

Because ${inf}_{\gamma ∼II\left(P_{L},P_{U}\right)}$ cannot be solved directly, we can learn from the generation method of the discriminator loss in Gulrajani et al. (2017). First, according to Xiao et al. (2019), Equation 13 can be equivalent to

$$W\left(P_{L},P_{U}\right)=\frac{1}{Z}\underset{\|f{\|}_{L}≦Z}{sup}\left(E_{x∼P_{L}}\left[f\left(x\right)\right]-E_{x∼P_{U}}\left[f\left(x\right)\right]\right)$$ (14)

When the Lipschitz constant $\|f{\|}_{L}$ does not exceed the constant Z , its upper bound $E_{x∼P_{L}}\left[f\left(x\right)\right]-E_{x∼P_{U}}\left[f\left(x\right)\right]$ can be taken for all f that can meet the condition, and we can use a set of parameters θ to define a series of possible functions $f_{\theta }$ . Then, Equation 14 can be approximately solved as Equation 15:

$$K▪W\left(P_{L},P_{U}\right)\approx \underset{\theta :|f_{\theta }{|}_{L}≦Z}{max}\left(E_{x∼P_{L}}\left[f_{\theta }\left(x\right)\right]-E_{x∼P_{U}}\left[f_{\theta }\left(x\right)\right]\right)$$ (15)

Due to the strong fitting ability of a deep neural network, a series of $f_{\theta }$ is enough to highly approximate ${sup}_{\|f{\|}_{L}≦Z}$ in Equation 14. So far, we can construct a discriminator network containing parameter θ within the limit of not more than under the condition of a certain range (−Q,Q) to ensure that Equation 16 holds true.

$$L_{W}\left(x\right)=E_{x∼P_{L}}\left[f_{\theta }\left(x\right)\right]-E_{x∼P_{U}}\left[f_{\theta }\left(x\right)\right]$$ (16)

The smaller the value of $L_{W}$ is, the smaller the Wasserstein distance representing the distribution of the instance is, which means that the instance is more representative and the model training is better. We denote fnail as this discriminator network. The loss is shown in Equation 17:

$$L=\underset{x\in D_{l}}{\sum }\left(L_{\mathrm{img}}\left(x\right)+L_{\mathrm{mil}}\left(x\right)\right)-\underset{x\in D_{u}}{\sum }\beta \left(▪L_{\mathrm{dis}}\left(x\right)+L_{W}\left(x\right)\right)$$ (17)

#### 2.5.3 Efficient active learning

Because the data with image-level annotation need annotation are expensive, the labeled samples in active learning are not sufficient to train a deep neural network because most unlabeled samples are usually ignored in active learning. It is difficult to obtain proper feature representation by fine-tuning the deep neural network using these few information samples. Therefore, we introduce a more efficient active learning method, namely, semi-supervised active learning (Wang et al., 2017; Li et al., 2021). Specifically, a few images with rich information help train more powerful classifiers, while most high-confidence images help learn more distinctive feature representations. On the one hand, although the number of labeled data is small, the most uncertain unlabeled samples usually have a great potential impact on the classifier. Selecting and labeling them in training can help to develop a better decision boundary for the classifier. On the other hand, although the performance of the classifier cannot be significantly improved, the unlabeled samples with high confidence are close to the labeled samples in the feature space of a deep neural network. The practice of pseudo-labeling and incorporating them into the training set can improve the generalization performance of modularity and is conducive to the low-density separation between categories (Zou et al., 2019; Wei et al., 2021).

Because the uncertainty between the image and the instance has been aligned, we select the top $2R_{n}$ samples with the highest uncertainty and select the top $R_{n}$ most representative instances from them. Then, we average the uncertainty of these instances to get the final image information degree. According to the ranking of image information degree, we select 10% of the images with the highest information each time to request experts for image-level annotation. Then, we pseudo-label the samples with the lowest information. It is worth noting that the pseudo-annotation technology of semi-supervised learning is similar to active learning. They must both learn image information, but the former is based on model-based image annotation, and the latter requires expert participation. We combine semi-supervised pseudo-labeling methods, make full use of unlabeled data, and build an efficient active learning model based on more training samples of the model to improve model stability and robustness. We use a dynamic threshold decay mechanism to enhance pseudo-label reliability as the model learns. Specifically, we choose the sample with the lowest information from the unmarked pool, and its image uncertainty value is less than the threshold δ , thus avoiding the introduction of noise from low-confidence pseudo labels. Then, we assign pseudo-labels with explicit predictions. Pseudo-label $y_{\mathrm{pse}}$ is defined as Equation 18:

$$y_{\mathrm{pse}}=argmax\left(y_{cr}\right)$$ (18)

It is worth noting that the image will be pseudo-marked only when the uncertainty of the image is less than δ . With the incremental learning process, the classification ability of the classifier is improved. In order to ensure the reliability of pseudo-labeled image sample selection, we update the threshold at the end of each active learning iteration a through the Equation 19: a through the following formula:

$$\delta =\left\{\begin{array}{cc} \delta_{0},\; & a=0\\ \delta -dr*a,\; & a>0 \end{array}\right.$$ (19)

where $\delta_{0}$ is the initial threshold and dr is the threshold decay rate.

### 2.6 The implementation

The proposed model is implemented using PyTorch (pytorch.org), and the objective function is minimized by using the AdmaW algorithm. We used an NVIDIA RTX3090 graphics card with 24 G memory. Parameters are set as follows: $K=100,A=10,epoch=20,\delta_{0}=0.01,dr=0.001,Q=0.01,Rn=6,C=2,T=2$ . The basic implementation code of this work can be found on GitHub at https://github.com/Lsx0802/AUW.

## 3 Experimental results

### 3.1 Experimental setup and evaluation metric

In order to assess the performance of the proposed method and conduct an objective comparison with other related methods, we annotate all tooth marks in tongue images at the image and instance levels in advance. Meanwhile, different proportions of labels were assigned in specific experiments to simulate the active learning process of interacting with doctors and objectively compared with other methods. Specifically, because only a few studies only used CNN as a feature extractor (Li et al., 2018; Wang et al., 2020; Zhou et al., 2022; Weng et al., 2021), we use Resnet34 (Wang et al., 2020) as the backbone for fair comparison in all the methods. The clinical screening data set contains 401 images of tooth-marked tongues and 707 images of non-tooth-marked tongues. We randomly used 108 of 1,108 images as an independent test set $D_{t}$ , containing 68 non-tooth-marked tongues and 40 tooth-marked tongues, and the remaining 1,000 images were used as the training set. We repeated the training and testing 10 times and evaluated the performance of the model by evaluating the average value of the indicators. For image-level tooth-marked tongue recognition, we used Accuracy, Precision, Recall, and F1 scores to evaluate the performance of the models. Clinically, patients with tooth-marked tongues need further treatment, so we hope the model has a higher Recall value under similar accuracy conditions. The following is a detailed description. TP represents True Positives, TN represents True Negatives, FP represents False Positives, and FN represents False Negatives.

Accuracy reflects the percentage of correct predictions made by the model over the entire dataset. It is calculated by the Equation 20:

$$\text{Accuracy}=\frac{TP+TN}{TP+TN+FP+FN}$$ (20)

Precision represents the proportion of all samples predicted by the model to be in the positive category that are actually in the positive category. It is calculated by Equation 21:

$$\text{Precision}=\frac{TP}{TP+FP}$$ (21)

Recall, also known as the sensitivity or true-positive rate, measures the proportion of samples that the model correctly identifies as being in the positive category of all samples that truly belong in the positive category. It is calculated as Equation 22:

$$\text{Recall}=\frac{TP}{TP+FN}$$ (22)

F1 score (F1-Score) is the reconciled average of precision and recall and is often used to balance the trade-off between the two. It is particularly adaptable to the problem of category imbalance. It is calculated by Equation 23:

$$\text{F}1-\text{Score}=2\times \frac{\text{Precision}\times \text{Recall}}{\text{Precision}+\text{Recall}}$$ (23)

### 3.2 Performance comparison with different methods

**FIGURE 4:**
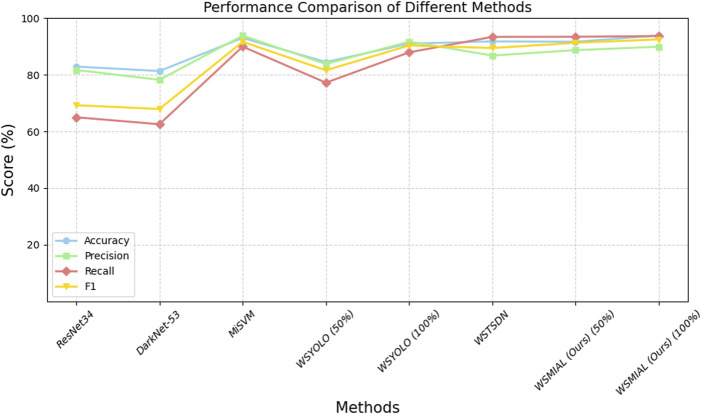
Performance comparison of different methods of tooth-marked tongue recognition.

Current tooth-marked tongue recognition methods (Li et al., 2018; Wang et al., 2020; Zhou et al., 2022; Weng et al., 2021) all require 100% image-level annotation, while the object detection-based methods (Li et al., 2018; Zhou et al., 2022; Weng et al., 2021) require further instance-level annotation. In order to obtain better detection performance (Li et al., 2018; Wang et al., 2020; Zhou et al., 2022), the tongue body must be segmented at the pixel level in advance in order to eliminate the interference of the background area outside the tongue. From the performance comparison in Table 1 and Figure 4, it can be seen that Li et al. (2018) have the top performance of tooth-marked tongue recognition among the above methods but also require the highest annotation cost. By comparison, our method can match the performance of Li et al. (2018) with only 50% image-level annotation without instance-level and pixel-level annotation. Moreover, after active learning completes 100% annotation of the image, the tooth-marked tongue recognition performance can slightly exceed the results of Li et al. (2018). This is because our active learning strategy based on instance uncertainty and diversity can make the model obtain a better decision boundary for tooth-marked tongue recognition. In addition, the pseudo-annotation method can make the active learning model with small data more robust.

**TABLE 1:** Performance comparison of different methods for tooth-marked tongue recognition (%).

Method	Annotation			Accuracy	Precision	Recall	F1
	**Image**	**Instance**	**Pixel**				
ResNet34 (Wang et al., 2020)	100%	-	100%	82.90	81.70	64.99	69.25
DarkNet-53 (Redmon and Farhadi, 2018)	100%	-	100%	81.32	78.22	62.53	67.91
MISVM (Li et al., 2018)	100%	100%	100%	93.03	93.84	89.95	91.66
WSYOLO (Weng et al., 2021)	100%	50%	-	84.50	83.78	77.21	81.59
WSYOLO (Weng et al., 2021)	100%	100%	-	90.99	91.71	87.92	90.36
WSTDN (Zhou et al., 2022)	100%	-	100%	91.80	86.80	93.39	89.45
WSMIAL (Ours)	50%	-	-	91.66	88.71	93.42	91.30
WSMIAL (Ours)	100%	-	-	93.88	89.90	93.70	92.50

### 3.3 Ablation study of multi-instance active learning

**FIGURE 5:**
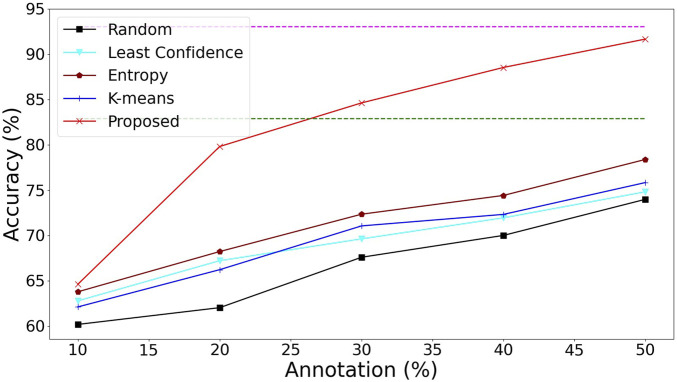
Accuracy of tooth-marked tongue recognition with different query strategies under active learning. The green dashed line represents the performance of ResNet34 (Wang et al., 2020), and the purple dashed line represents the performance of MISVM Li et al. (2018).

In terms of query strategy in active learning, we compare the proposed query strategy with baseline random sampling (random) and common active learning strategies based on instance filtering, such as sampling based on least confidence (Guo et al., 2017), sampling based on uncertainty (entropy) (Lakshminarayanan et al., 2017), and unsupervised clustering sampling based on diversity (K-means) (Ash et al., 2019). As shown in Figure 5, the sampling based on uncertainty outperforms random, minimum confidence (least confidence), and clustering (K-means). Under the same detection model (Section 2.4 WSL module), the entropy method has the best performance among the common active learning strategies, but they all fail to surpass the performance of WSL’s backbone Resnet34 under 100% annotation. The methods of deterministic learning and representative learning of examples are targeted to solve the problems of weakly supervised detection of background noise and lack of representativeness of examples and make full use of unlabeled data, thus surpassing other common active learning strategies.

**FIGURE 6:**
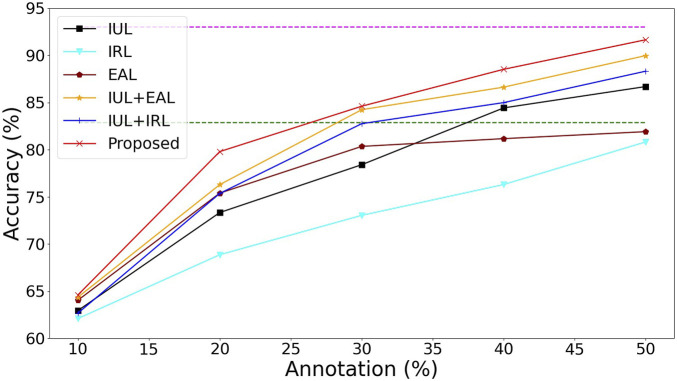
Ablation performance of the proposed model with the accuracy of tooth-marked tongue recognition. The green dashed line represents the performance of ResNet34 (Wang et al., 2020), and the purple dashed line represents the performance of MISVM Li et al. (2018).

As shown in Figure 6, IUL represents the uncertainty of our alignment instances and images, IRL represents that we use the Wasserstein distance to learn the diversity of instances, and EAL represents the introduction of the pseudo-labeling process. Because we have excluded noisy instances by aligning instances to image uncertainty (IUL), the performance of the model is further improved, outperforming direct classification with 100% image-level annotations while requiring only 40% of image-level annotations way of ResNet34 Wang et al. (2020). Our approach (Proposed) outperforms ResNet34’s approach when only 30% image-level annotations are required. In the case where only 50% image-level annotations are required, our method can achieve almost the same performance as the fully annotated MISVM method Li et al. (2018). Combined with the results in Table 1, our model outperforms the MISVM method when it is finally trained to 100% image-level annotation (Table 1), which demonstrates that our method can greatly reduce the annotation cost while maintaining a high level of performance.

**FIGURE 7:**
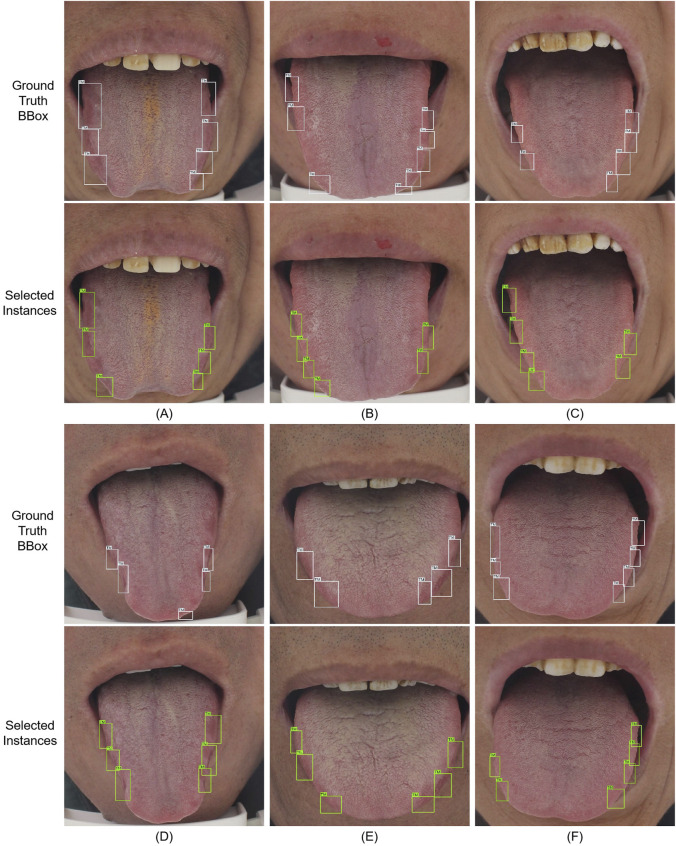
Ground-truth labels of the tooth mark location boxes and examples selected by our method. **A–D** are the images selected by the model in the second and fifth rounds of active learning to request expert annotation respectively. **E, F** are the samples selected in the fifth round of pseudo-labeling process of the model.

**FIGURE 8:**
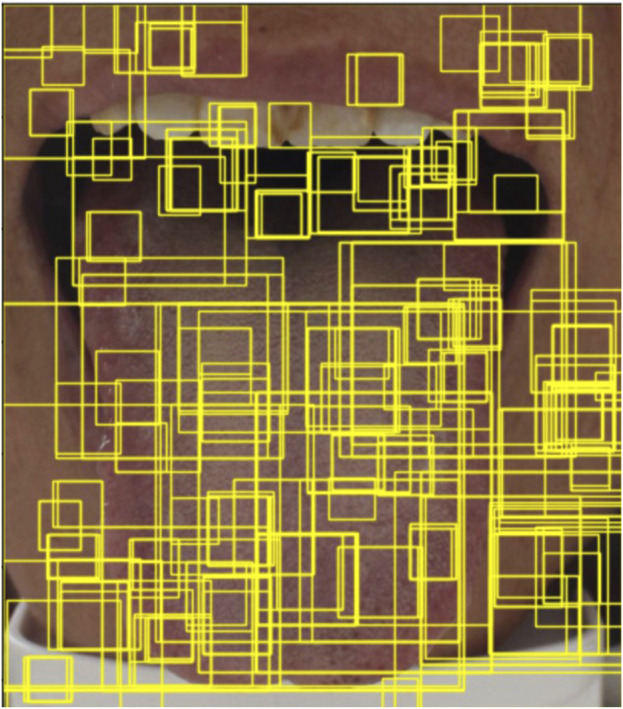
An example generated by the selective search method Van de Sande et al. (2011) based on the common method of object detection. The yellow bounding boxes shown in the figure represent the object proposals generated by the selective search algorithm.

Finally, we visualize the selected instances to confirm that our method can improve the accuracy of image recognition through instance information and the effect of weakly supervised localization to assist clinical diagnosis. As shown in Figure 7, our method can reduce the background noise of the instances shown in Figure 8 and highlight the most informative instances by aligning the uncertainty of instances and images and learning from the diversity of instances. A–B and C–D are the images selected by the model in the second and fifth rounds of active learning to request expert annotation, respectively. In particular, as shown in A, the model is not sensitive to large tooth marks, which may be caused by the fact that we do not use multi-scale detection methods like the common one-stage method. In B and D, the model fails to detect the tongue tip. This may be due to the fact that most of the teeth marks on the tongue are located on both sides of the tongue (Zhou et al., 2022). The model identifies the instances in the upper left corner of C and D as informative regions, and these regions were also identified by later experts as earlier annotation errors (as we introduced in Section “Experiment setup and evaluation metric,” we first label all and then simulate the process of requesting experts to label), the difficult areas where these experts will make mistakes are the areas that need the most attention of the model. E and F are the samples selected in the fifth round of the pseudo-labeling process of the model. It can be clearly seen that the model has made more accurate discrimination on the tooth mark area of these images, so the final information of these images is the lowest, and the pseudo-labels are added to enrich the training data.

## 4 Discussion

In this work, we propose an efficient multi-instance active learning model to recognize tooth-marked tongues. This model is based on incomplete and inaccurate supervision within the weakly supervised learning framework. Compared with the previous tooth-marked tongue recognition methods (Li et al., 2018; Wang et al., 2020; Zhou et al., 2022; Weng et al., 2021), the proposed method solves the problem of inaccurate supervision based on multi-instance learning and does not need a large number of instance-level annotation. The proposed method adopts the one-stage detection method and does not need the pixel-level annotation of tongue pre-segmentation. In addition, we build an active learning framework, which is not available in the previous tooth-marked recognition methods. We use the most informative data to request expert annotation so that the model can better represent the tooth mark distribution and reduce the amount of image-level annotation. Specifically, we construct the consistency of instance uncertainty and image uncertainty to reduce the noise instances in tooth mark detection. We further screened the toothmark instances based on the diversity of instances, and were able to extract more representative instances.

For active learning, previous studies have used the uncertainty and diversity of images to select image samples (Wang et al., 2018; Ash et al., 2019), combined the pseudo-tagging technology of semi-supervised learning with active learning (Wang et al., 2017; Zhang et al., 2022), and combined multi-instance target detection with active learning (Yuan et al., 2021). However, the proposed method is different. We used only image-level annotation based on weakly supervised multi-instance learning and the uncertainty and diversity of samples to screen the information of image samples and construct an efficient framework of active learning by using pseudo-labeling technology. Compared with the training model with 100% all-labeled data, the accuracy results of 30%–50% samples are also competitive. Note that this is under the premise of active learning of traditional Chinese medicine data with small samples; 50% of the data of 1,108 cases is actually not much and is difficult to compare with 20% of the data of 7,295 cases of active learning of mainstream medical data (Zhang et al., 2022). Our method of constructing image information based on instance information captures fine-grained information, enabling active learning models to be effective with small datasets.

Although our proposed method is designed to solve the problem of excessive annotation of instances in tooth-marked tongue recognition, it can be applied to many other applications in the field of active detection that require much instance-level annotation, such as active detection of video streams (Zhu et al., 2020) or the field of instance-level feature extraction based on image-level annotation only, such as whole slide image (WSI) classification (Shao et al., 2021). The study reported that one of the challenges of video detection is the inconsistent cognition of labels and the time-consuming and laborious labeling (Zhu et al., 2020). In the future, video detection technology can be based on weak supervision or self-supervision. In addition, a WSI image is generally large and difficult to obtain and, therefore, very difficult to label accurately (Shao et al., 2021). In the case of small instances, our method can reduce the instance-level and image-level annotation and may not significantly decrease the detection and classification performance. In addition, the query strategy based on the uncertainty and diversity of examples can better solve the problem of instance diversity.

There are some studies that need to be improved in this work. First, there is some correlation information between the instance tooth marks of the same tongue. Previous studies (Shao et al., 2021; Schölkopf et al., 2007) have shown that the mining of multi-instance correlation information can further improve detection performance. Therefore, it is possible to improve the accuracy of the model by using the correlation between tooth marks. Then, our method is based on the model of object detection, but most object detection models are based on multi-scale to obtain joint accuracy. In this work, we select the appropriate (shallow) scale to obtain the detection accuracy. Because the tooth mark is generally small on the tongue and accounts for a small proportion of the whole picture, a shallow scale can have good detection performance for small targets. However, the multi-scale method has been demonstrated to have better detection performance in the field of object detection, so we will consider how to further improve the performance based on multi-scale object detection. In addition, we can strengthen the original feature representation through feature reconstruction (Chen et al., 2024). This will enable the model to better capture the key features of tooth-marked tongues. After feature extraction, a voting integration method (Bao et al., 2024) can be employed. By combining the predictions of multiple weakly supervised classifiers through weighted voting, the influence of noisy labels can be mitigated. This process enhances the model’s robustness and accuracy. Furthermore, there are not only tooth marks on the tongue but also cracks, ecchymosis, congestion, and other diseases, and we have not considered these additional symptoms in this work. In future research, we will focus on conducting specialized multi-class, multi-label, and multi-center studies on tongue images.

## 5 Conclusion

In this study, we proposed a weakly supervised active learning tooth-marked tongue detection model with only a few image-level labels. Experimental results showed that the proposed method is effective with only a small amount of image-level annotation, and its performance is comparable to that of image-level annotation, instance-level annotation, and pixel-level annotation, which require many tooth markers. Our method significantly reduces the annotation cost of the binary classification task of traditional Chinese medicine tooth mark recognition.

## Data Availability

The original contributions presented in the study are included in the article/supplementary material; further inquiries can be directed to the corresponding author.

## References

[B1] Andrews S. Tsochantaridis I. Hofmann T. (2002). “Support vector machines for multiple-instance learning,” in Advances in Neural Information Processing Systems. NIPS.

[B2] Ash J. T. Zhang C. Krishnamurthy A. Langford J. Agarwal A. (2019). Deep batch active learning by diverse, uncertain gradient lower bounds. CoRR abs/1906, 03671. 10.48550/arXiv.1906.03671

[B3] Bao W. Liu Y. Chen B. (2024). Oral_voting_transfer: classification of oral microorganisms’ function proteins with voting transfer model. Front. Microbiol. 14, 1277121. 10.3389/fmicb.2023.1277121 38384719 PMC10879614

[B4] Bilen H. Vedaldi A. (2016). “Weakly supervised deep detection networks,” in 2016 IEEE Conference on Computer Vision and Pattern Recognition (CVPR) (IEEE), 2846–2854. 10.1109/CVPR.2016.311

[B5] Chen B. Li N. Bao W. (2024). Clpr_in_ml: cleft lip and palate reconstructed features with machine learning. Curr. Bioinforma. 20, 179–193. 10.2174/0115748936330499240909082529

[B6] David Zhang B. Z. Zhang H. (2017). Tongue image analysis. 10.1007/978-981-10-2167-1

[B7] Fu J. Zheng H. Mei T. (2017). “Look closer to see better: recurrent attention convolutional neural network for fine-grained image recognition,” in 2017 IEEE Conference on Computer Vision and Pattern Recognition (CVPR) (IEEE), 4476–4484. 10.1109/CVPR.2017.476

[B8] Girshick R. (2015). Fast r-cnn. IEEE International Conference on Computer Vision ICCV, 1440–1448. 10.1109/ICCV.2015.169

[B9] Gulrajani I. Ahmed F. Arjovsky M. Dumoulin V. Courville A. C. (2017). Improved training of wasserstein gans. CoRR abs/1704, 00028. 10.48550/arXiv.1704.00028

[B10] Guo C. Pleiss G. Sun Y. Weinberger K. Q. (2017). “On calibration of modern neural networks,” in Proceedings of the 34th international conference on machine learning. (PMLR), vol. 70 of Proceedings of machine learning research. Editors Precup D. Teh Y. W., 1321–1330.

[B11] He K. Zhang X. Ren S. Sun J. (2016). “Deep residual learning for image recognition,” in 2016 IEEE Conference on Computer Vision and Pattern Recognition (CVPR), 770–778. 10.1109/CVPR.2016.90

[B12] Hsu Y. C. Chen Y. C. Lo L. C. Chiang J. Y. (2011). “Automatic tongue feature extraction,” in 2010 international computer symposium (ICS2010). 10.1109/COMPSYM.2010.5685377

[B13] Lakshminarayanan B. Pritzel A. Blundell C. (2017). Simple and scalable predictive uncertainty estimation using deep ensembles.

[B14] Li X. Zhang Y. Cui Q. Yi X. Zhang Y. (2018). Tooth-marked tongue recognition using multiple instance learning and cnn features. IEEE Trans. Cybern. 49, 380–387. 10.1109/TCYB.2017.2772289 29994570

[B15] Li Y. Fan B. Zhang W. Ding W. Yin J. (2021). Deep active learning for object detection. Inf. Sci. 579, 418–433. 10.1016/j.ins.2021.08.019

[B16] Lo L. C. Chen Y. F. Chen W. J. Cheng T. L. Chiang J. Y. (2012). The study on the agreement between automatic tongue diagnosis system and traditional Chinese medicine practitioners. Evidence-Based Complementray Altern. Med. 2012, 505063. 10.1155/2012/505063 PMC3424603 22924055

[B17] Lu J. Liu M. Chen H. (2023). “Prtmtm: *a priori* regularization method for tooth-marked tongue classification,” in 2023 IEEE International Symposium on Circuits and Systems (ISCAS), Monterey, CA, USA (IEEE), 1–5. 10.1109/ISCAS46773.2023.10181870

[B18] Redmon J. Divvala S. Girshick R. Farhadi A. (2016). “You only look once: unified, real-time object detection,” in 2016 IEEE conference on computer vision and pattern recognition (CVPR), 779–788. 10.1109/CVPR.2016.91

[B19] Redmon J. Farhadi A. (2018). Yolov3: an incremental improvement. CoRR abs/1804.02767. 10.48550/arXiv.1804.02767

[B20] Schölkopf B. Platt J. Hofmann T. (2007). Multi-instance multi-label learning with application to scene classification, 1609–1616.

[B21] Selvaraju R. R. Cogswell M. Das A. Vedantam R. Parikh D. Batra D. (2017). “Grad-cam: visual explanations from deep networks via gradient-based localization,” in 2017 IEEE International Conference on Computer Vision (ICCV), Venice, Italy (IEEE), 618–626. 10.1109/ICCV.2017.74

[B22] Shao Q. Li X. Fu Z. (2014). “Recognition of teeth-marked tongue based on gradient of concave region,” in 2014 International Conference on Audio, Language and Image Processing, Shanghai, China (IEEE), 968–972. 10.1109/ICALIP.2014.7009938

[B23] Shao Z. Bian H. Chen Y. Wang Y. Zhang J. Ji X. (2021). Transmil: transformer based correlated multiple instance learning for whole slide image classication. CoRR abs/2106, 00908. 10.48550/arXiv.2106.00908

[B24] Sun Y. Dai S. Li J. Zhang Y. Li X. (2019). Tooth-marked tongue recognition using gradient-weighted class activation maps. Future Internet 11, 45. 10.3390/fi11020045

[B25] Tan W. Chang D. Li J. He D. (2023). “Tooth-marked tongue recognition based on wavelet transform and feature fusion,” in 2023 IEEE 6th Information Technology,Networking,Electronic and Automation Control Conference, Chongqing, China (IEEE), 6, 1274–1280. 10.1109/ITNEC56291.2023.10082166

[B26] Van de Sande K. E. Uijlings J. R. Gevers T. Smeulders A. W. (2011). “Segmentation as selective search for object recognition,” in 2011 international conference on computer vision (IEEE), Barcelona, Spain (IEEE), 1879–1886. 10.1007/s11263-013-0620-5

[B27] Villani C. (2009). Optimal transport: Old and new. grundlehren der mathema-tischen wissenschaften. Springer.

[B28] Wang K. Zhang D. Li Y. Zhang R. Lin L. (2017). Cost-effective active learning for deep image classification. IEEE Trans. Circuits Syst. Video Technol. 27, 2591–2600. 10.1109/TCSVT.2016.2589879

[B29] Wang M. Fu K. Min F. (2018). “Active learning through two-stage clustering,” in 2018 IEEE International Conference on Fuzzy Systems, Rio de Janeiro, Brazil (FUZZ-IEEE), 1–7. 10.1109/FUZZ-IEEE.2018.8491674

[B30] Wang X. Liu J. Wu C. Liu J. Chen J. Chen Y. (2020). Artificial intelligence in tongue diagnosis: using deep convolutional neural network for recognizing unhealthy tongue with tooth-mark. Comput. Struct. Biotechnol. J. 18, 973–980. 10.1016/j.csbj.2020.04.002 32368332 PMC7186367

[B31] Wei C. Sohn K. Mellina C. Yuille A. Yang F. (2021). “Crest: a class-rebalancing self-training framework for imbalanced semi-supervised learning,” in 2021 IEEE/CVF conference on computer vision and pattern recognition (CVPR), 10852–10861. 10.1109/CVPR46437.2021.01071

[B32] Weng H. Li L. Lei H. Luo Z. Li C. Li S. (2021). A weakly supervised tooth-mark and crack detection method in tongue image. Concurrency Comput. Pract. Exp. 33. 10.1002/cpe.6262

[B33] Xiao H. Herman M. Wagner J. Ziesche S. Etesami J. Linh T. H. (2019). Wasserstein adversarial imitation learning. CoRR abs/1906.08113. 10.48550/arXiv.1906.08113

[B34] Yuan T. Wan F. Fu M. Liu J. Xu S. Ji X. (2021). “Multiple instance active learning for object detection,” in 2021 IEEE/CVF conference on computer vision and pattern recognition (CVPR), 5326–5335. 10.1109/CVPR46437.2021.00529

[B35] Zhang M. Wang Y. Ma X. Xia L. Yang J. Li Z. (2020). “Wasserstein distance guided adversarial imitation learning with reward shape exploration,” in 2020 IEEE 9th Data Driven Control and Learning Systems Conference (DDCLS), Liuzhou, China (IEEE), 1165–1170. 10.1109/DDCLS49620.2020.9275169

[B36] Zhang W. Zhu L. Hallinan J. Zhang S. Makmur A. Cai Q. (2022). “Boostmis: boosting medical image semi-supervised learning with adaptive pseudo labeling and informative active annotation,” in Proceedings of the IEEE/CVF conference on computer vision and pattern recognition.

[B37] Zhou J. Li S. Wang X. Yang Z. Hou X. Lai W. (2022). Weakly supervised deep learning for tooth-marked tongue recognition. Front. Physiology 13, 847267. 10.3389/fphys.2022.847267 PMC9039050 35492602

[B38] Zhou Z.-H. (2017). A brief introduction to weakly supervised learning. Natl. Sci. Rev. 5, 44–53. 10.1093/nsr/nwx106

[B39] Zhu Y. Li X. Liu C. Zolfaghari M. Xiong Y. Wu C. (2020). A comprehensive study of deep video action recognition. CoRR abs/2012, 06567. 10.48550/arXiv.2012.06567

[B40] Zitnick C. L. Dollár P. (2014). “Edge boxes: locating object proposals from edges,” in Computer vision – eccv 2014. Editors Fleet D. Pajdla T. Schiele B. Tuytelaars T. (Cham: Springer International Publishing), 391–405.

[B41] Zou Y. Yu Z. Liu X. Kumar B. V. K. V. Wang J. (2019). “Confidence regularized self-training,” in 2019 IEEE/CVF international conference on computer vision (ICCV), 5981–5990. 10.1109/ICCV.2019.00608

